# Grape Seed Proanthocyanidin Extract Prevents Ovarian Aging by Inhibiting Oxidative Stress in the Hens

**DOI:** 10.1155/2018/9390810

**Published:** 2018-01-09

**Authors:** Xingting Liu, Xin Lin, Yuling Mi, Jian Li, Caiqiao Zhang

**Affiliations:** Department of Veterinary Medicine, College of Animal Sciences, Zhejiang University, No. 866 Yuhangtang Road, Hangzhou 310058, China

## Abstract

Oxidative stress is an important inducement in ovarian aging which results in fecundity decline in human and diverse animals. As a potent antioxidant, grape seed proanthocyanidin extract (GSPE) was investigated to ameliorate chicken ovarian aging in this study. Firstly, ovarian antioxidant capacity of hens at different ages (90, 150, 280, and 580 days old) was compared to elucidate its age-related changes. Subsequently, a D-gal-induced (2.5 mg/mL) aging ovarian model was established and the cultured ovarian tissues were treated with GSPE at 5 *μ*g/mL for 72 h to evaluate the putative attenuating effects of GSPE on ovarian aging. Meanwhile, ovaries of D280 (young) and D580 (old) were treated with GSPE for 72 h in culture to verify the protective effects of GSPE on natural aging ovary. The results showed that GSPE could rescue the antioxidant capacity decline by increasing the antioxidase activities and their gene expression in either D-gal-induced or natural aging ovaries. Moreover, GSPE could maintain the homeostasis between cell proliferation and apoptosis in the D-gal-induced and natural aging ovaries, as well as alleviate D-gal-induced nucleus chromatin condensation in the ovarian granulosa cells. In conclusion, GSPE treatment can effectively prevent the ovarian aging process in hens by reducing oxidative stress.

## 1. Introduction

In mammals, female reproductive capacity is negatively correlated with age, and the female reproductive system is more rapid to show overt signs of physiologic aging than other body systems [1]. Female reproductive aging is known to trigger a series of molecular alterations that could cause aneuploidy, miscarriages, birth defects, and infertility [2, 3]. Ovarian aging is thought to be dominated by a gradual decrease in both the quantity and the quality of the oocytes residing within the follicles with increasing age [4, 5]. These decreases lead to the decline in the duration of ovarian functions. Similar to mammals, the poultry also undergoes age-related decline in fecundity. Fast decline appears in the egg production of the laying hens after 480 days of age due to ovarian aging, thereby leading to reduced egg production and commercial value of the laying hens. Hence, it is necessary to explore the mechanisms of the deterioration of the laying performance and its countermeasures during the aging process.

One of the major factors that cause ovarian aging is oxidative stress [6]. The oxidative stress induced by accumulated reactive oxygen species (ROS) levels is indicated to be one of the dominant mechanisms underlying ovarian aging [7, 8]. In the living organisms, physiological levels of ROS are appreciated to maintain the normal conditions such as signal transduction and redox regulation, while high levels of ROS could induce oxidative damage [8, 9]. In the normal cells, there is a complex antioxidant defense system which could scavenge ROS and maintain the redox state of diverse cells. The antioxidant defense system consists of antioxidant enzymes such as superoxide dismutase (SOD), catalase (CAT), glutathione peroxidase (GSH-Px), and glutathione S-transferase (GSH-ST) and biological antioxidants such as glutathione (GSH) and vitamins C and E [7, 10]. However, the balance between ROS generation and scavenging would be disrupted due to decreasing levels of the antioxidants during the aging process, then causing oxidative stress [11]. Therefore, the balance between the antioxidants and oxidants is regarded as an important parameter that could reflect the antioxidant capacity of the tissues or organs.

Previous studies demonstrated that ROS accumulation in ovarian tissues can lead to the granulosa cell apoptosis and atresia of the antral follicles in rats [12]. In mice, oxidative stress inhibits growth and induces atresia of the antral follicles [13]. Growing evidences have shown that high level of ROS is associated with age-related decline in female reproduction [14–16]. Numerous studies have demonstrated oxidative stress in the aging process. In women, ROS scavenging efficiency in the follicular fluid undergoes a significant decrease during aging process as a result of the decline in the activities of several antioxidant enzymes [17]. In mice, oxidative stress was detected in the aging adipose [18] and brain [19]. However, the probable changes in levels of antioxidants in chicken ovary with aging remain unknown.

The establishment of aging models is a common method to investigate the fundamental mechanisms of aging. D-galactose (D-gal) is a reducing sugar that can generate advanced glycation end products (AGEs) in its oxidative metabolism *in vivo* [20]. In mice, oversupply of D-gal induces changes that are similar to natural aging, such as increased oxidative stress [21], cognitive dysfunction [22], and neurodegeneration [23]. The D-gal-induced aging model has been widely used for the exploration of aging mechanism and the screening for antiaging substances. Studies on rodents demonstrated that D-gal could induce oxidative stress in the brain [24], liver [25], and ovary [26].

Ovarian aging is considered to be one of the highest risk factors that lead to the decline of ovarian functions. Hence, the exploration of the antiaging measures is essential to retard ovarian aging process. In recent years, many natural plant extractions such as resveratrol [27] and hesperidin [28] were used for reducing oxidative stress in ovarian tissues in order to maintain the normal function of the ovary. Grape seed proanthocyanidin extract (GSPE) is a kind of phenol compounds that exist in fruits, vegetables, nuts, seeds, wine, and tea. It has been shown to provide excellent protection against free radicals and oxidative stress-mediated tissue injury [29, 30]. However, its antioxidant role has not been well understood in senescent ovaries of the laying hens.

In the present study, the antioxidant status of hen ovaries at four different stages (90, 150, 280, and 580 days old) was analyzed and compared to elucidate the relationship between oxidative stress and ovarian aging. Subsequently, a D-gal-induced aging ovary model was established to evaluate the protection of GSPE on ovarian aging process *in vitro*. Meanwhile, ovaries from hens at 280 days (D280) and 580 days (D580) were treated with GSPE for 72 h *in vitro* to verify the effects of GSPE on the antioxidant status in the natural aging process. The results will expand the knowledge about retarding ovarian aging of laying hens and extending egg production in the elder layers for poultry production.

## 2. Materials and Methods

### 2.1. Chemicals and Reagents

GSPE power was obtained from Tianjin Jianfeng Natural Product R&D Co. Ltd. (purity ≥ 95%, Tianjin, China). D-gal power for cell culture was purchased from Aladdin Industrial Co. (Shanghai, China; purity ≥ 95%). Dulbecco's Modified Eagle Medium/F12 (DMEM-F12), penicillin, and streptomycin were purchased from Hyclone-Pierce (Fremont, CA, USA). Primary antibodies against proliferation cell nuclear antigen (PCNA), *β*-actin, BCL-xL, and Bax were purchased from Abcam (Cambridge, Massachusetts, USA). Antibodies against cyclin D1 (CCND1) and cyclin-dependent kinase 2 (CDK2) were purchased from Boster Biological Technology Co. Ltd. (Wuhan, China). Biochemical parameter kits were obtained from Nanjing Jiancheng Bioengineering Institute (Nanjing, China). All other chemicals were analytical reagents.

### 2.2. Animals and Tissue Collection

Hyline brown hens were raised in a local commercial farm and subjected to conventional feeding management conditions. All animal experiments were performed in accordance with the Animal Care and Use Committee on the Ethics of Animal Experiments of Zhejiang University. Sample collection was performed from D90, D150, D280, and D580 hens that reflected four different laying stages, that is, before laying, early laying, peak laying, and later laying periods. Hens were slaughtered by cervical bleeding postanaesthesia, and ovaries without follicles over 1 mm in diameter were immediately snap-frozen in liquid nitrogen for the analysis of the biochemical parameters and RNA extraction.

### 2.3. Organ Culture and Treatment

For organ culture, ovaries of the D90 pullets were placed in ice-cold DMEM-F12 supplemented with 100 U/mL penicillin-streptomycin. Ovarian cortical blocks (1-2 cm^3^) were dissected from the surface of the ovaries. Each of the blocks was placed into a 24-well plate containing 500 *μ*L complete DMEM-F12 supplemented with 5% chicken serum, 10 *μ*g/mL insulin, 5 *μ*g/mL transferrin, and 30 nM selenite (Sigma-Aldrich, St. Louis, MO, USA). D-gal and GSPE power was dissolved in DMEM-F12. All of the cultures were maintained in a water-saturated atmosphere of 95% air and 5% CO_2_ at 38.5°C, and the medium was replaced every 24 h. After 48 h treatment, bromodeoxyuridine (BrdU, Sigma-Aldrich, St. Louis, CA, USA) was added into the complete medium at 20 *μ*g/mL. Before the formal experiment, the cultures were treated with D-gal from 1.25 mg/mL to 5 mg/mL to induce oxidative damage. Based on the evaluation of tissue morphology, cell proliferation, and apoptosis rates, the dose of 2.5 mg/mL D-gal was chosen as the optimal concentration in the subsequent experiments (Supplementary Figures 1–3). Likewise, four gradient concentrations of GSPE (from 0.05 to 50 *μ*g/mL) were screened for the optimal concentration under D-gal-induced oxidative damage. Based on the tissue morphology, cell proliferation, and apoptosis rates, 5 *μ*g/mL was selected as the optimal concentration in the following experiments (Supplementary Figures 4-5). The ovarian cortical blocks were divided randomly into four groups and were treated with D-gal (2.5 mg/mL), GSPE (5 *μ*g/mL), and D-gal + GSPE. After 72 h culture, the ovarian cortical tissues were fixed in 4% paraformaldehyde for morphological observation and fluorescence immunohistochemistry. The ovarian cortical tissues for biochemical analysis, qRT-PCR, and Western blot were cultured 72 h without BrdU incorporation. Moreover, ovarian cortex (follicle diameter < 1 mm) from D280 and D580 hens was dissected and cut into small fragments (1-2 cm^3^) and cultured for treatment with 5 *μ*g/mL GSPE for 72 h.

### 2.4. Morphological Observation

Ovaries from D90, D150, D280, and D580 hens were dissected and fixed in 4% neutral paraformaldehyde solution for up to 24 h at 4°C. The ovarian fragments and cultured ovarian cortical blocks were then dehydrated in a graded ethanol, then cleared in xylene and embedded in paraffin. The embedded tissue samples were sectioned at 5 *μ*m and mounted on slides. Hematoxylin and eosin (H&E) staining was performed using standard protocols.

### 2.5. Measurements of Oxidative Parameters

Ovarian tissues were homogenized in phosphate-buffered saline (PBS) and then centrifuged at 800*g* for 20 min at 4°C to obtain 10% tissue homogenate. The supernatants were used for the determination of total protein concentration and measurements of the oxidative parameters. Total protein concentration, the activity of SOD, GSH-Px, GSH-ST, CAT, and total antioxidant capacity (T-AOC) and the concentrations of GSH, malonaldehyde (MDA), and hydrogen peroxide (H_2_O_2_) were measured according to the manufacturer's instruction with kits (Nanjing Jiancheng Bioengineering Institute, Nanjing, China).

### 2.6. RT-PCR and qRT-PCR

Total RNA was extracted from ovarian tissues using TRIzol reagent (TaKaRa, Dalian, China) according to the manufacturer's protocol. The cDNA was synthesized using the RevertAid First Strand cDNA Synthesis Kit (Thermo Fisher Scientific, San Jose, CA, USA) following the manufacturer's instruction. The reverse transcription product was diluted 1 : 10 and then used as a cDNA template for qRT-PCR analysis. The qRT-PCR was carried out in an ABI 7500HT Real-Time PCR detection system (Applied Biosystems, Foster City, CA, USA) with the reaction volume of 20 *μ*L that contained 2 *μ*L cDNA template, 400 nM of each of the gene-specific forward and reverse primers, 0.4 *μ*L Rox reference dye II, 10 *μ*L SYBR Premix Ex Taq (TaKaRa, Shiga, Japan), and 6.8 *μ*L water. qRT-PCR conditions were as follows: 95°C for 10 min and then 40 cycles of 95°C for 30 s, 60°C for 34 s, and 72°C for 30 s. Comparisons of expression levels were determined by delta CT method normalized to *β*-actin. Sequences of the primers are provided in Table 1.

### 2.7. Immunofluorescence Staining

Paraffin-embedded ovarian tissue sections were deparaffinized and rehydrated. Antigen retrieval was performed in a 10 mM sodium citrate buffer solution (pH 6.0) for 20 min followed by 2 M HCl denaturation at 37°C for 30 min and neutralization by 0.1 M sodium tetraborate for 10 min at room temperature. Blocking was performed in 5% goat serum (Boster Biological Technology Co. Ltd., Wuhan, China) for 20 min at room temperature. Then, the tissue sections were incubated with mouse anti-BrdU monoclonal antibody (1 : 200, G3G4, DSHB, Iowa, USA) overnight at 4°C. After that, tissue sections were incubated with a 1 : 500 dilution of goat anti-mouse secondary antibody conjugated to TRITC (Invitrogen, Carlsbad, CA, USA) for 1 h at 37°C and counterstained with 4′,6-diamidino-2-phenylindole (DAPI, Sigma-Aldrich, St. Louis, CA, USA) for cell nuclei. Observations were performed under Olympus IX81 or confocal microscopy (Fluoview 300; Olympus, Tokyo, Japan). The number of the BrdU-positive cells (red) was counted and expressed as the percentage of the BrdU labeling cells with the total number of ovarian cells within the same fields (BrdU index).

### 2.8. TUNEL Analysis of Apoptosis

Apoptosis of cells was detected by a TUNEL Brightgreen Apoptosis Detection Kit (Vazyme Biotech Co. Ltd., Nanjing, China) according to the manufacturer's protocol. Tissue sections were counterstained with DAPI for 5 min. Five fields of each group were randomly selected for counting the number of the TUNEL-positive cells (green). The apoptosis rate was calculated as the percentage of the green labeling cells with the total number of ovarian cells (TUNEL index).

### 2.9. Western Blot Analysis

Ovaries were homogenized in RIPA that was supplemented with proteinase inhibitors. After centrifugation at 13000*g* for 20 min at 4°C, equal amounts of proteins were loaded and separated by 10% SDS-PAGE gel and thereafter transferred onto a polyvinylidene difluoride membrane. The membrane was blocked in 5% dry skim milk in 0.01 M PBS and 0.1% Tween 20 at room temperature for 2 h and then incubated overnight at 4°C with antibodies of rabbit against CCND1, CDK2, and BCL-xL (Boster Bioengineering Co. Ltd., Wuhan, China) and mouse against *β*-actin, Bax, and PCNA (Abcam, Cambridge, Massachusetts, USA), followed by incubation with the secondary horseradish peroxidase-conjugated goat anti-rabbit and anti-mouse antibodies (Boster Bioengineering Co. Ltd., Wuhan, China) for 1 h at room temperature.

### 2.10. Measurement of ROS

ROS was determined by a ROS Assay Kit (Nanjing, China). Briefly, after 72 h of culture, ovarian blocks were minced and digested in 0.25% trypsin and EDTA solution (E. Merk, Darmstadt, Germany) at 37°C for 5 min and then for 5 min with intermittent aspiration. The dispersed cells were filtered through a 300 gauze mesh and centrifuged for 10 min at 500*g*. The cell suspension was incubated with an oxidation-sensitive fluorescent probe (DCFH-DA, 10 *μ*M) in PBS for 1 h at 37°C. The cell suspension was centrifuged for 10 min at 1000*g* and washed three times with PBS. Florescence signal was detected using a Molecular Device SpectraMax M2 Microplate Reader (Sunnyvale, Silicon Valley, USA). The ROS level was expressed as the ratio of the fluorescent intensity to the total protein concentration of the cell suspension.

### 2.11. Transmission Electron Microscopy

The specimen of ovarian cortical blocks were fixed into 2.5% glutaraldehyde in PBS (0.1 M, pH 7.0) for 24 h at 4°C after 72 h of culture. The specimen were postfixed with 1% osmium tetroxide (OsO4) in PBS for 1.5 h at room temperature and then rinsed in PBS. The samples were dehydrated in a graded series of ethanol for 15 min at each step and then transferred to absolute acetone for 20 min. The samples were then infiltrated with 1 : 1 mixture of absolute acetone and the final Spurr resin mixture for 1 h at room temperature and were subsequently transferred to 1 : 3 mixture of absolute acetone and the final resin mixture for 3 h. After embedding in Araldite, the samples were sectioned in Leica EM UC7 ultramicrotome (Leica Microsystems GmbH Wetzlar, Germany) and the ultrathin sections (50 nm) were mounted on copper-coated grids. The ultrathin sections were stained with uranyl acetate and alkaline lead citrate for 5 to 10 min. The cell ultrastructure was observed using a transmission electron microscope (Tecnai G2 Spirit 120kV FEI Company, Hillsboro, USA).

### 2.12. Statistical Analysis

All experiments were repeated at least three times. Data were presented as the mean ± s.e. Analyses were performed with one-way ANOVA with post hoc Dunnett's test and independent samples *t*-test using SPSS 20.0 software (SPSS Inc., NY, USA) and GraphPad Prism 5 (GraphPad Software Inc., San Diego, CA, USA). Results were considered statistically significant at *P* < 0.05.

## 3. Results

### 3.1. Age-Related Changes in Ovarian Morphology and Laying Performance

**Figure 1:**
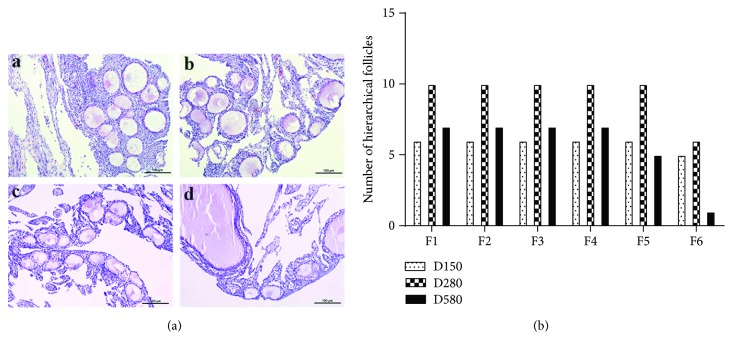
Ovarian histology and hierarchical follicle number in hens at different ages. (a) Representative morphology of ovarian tissues in hens aged 90 (A), 150 (B), 280 (C), and 580 (D) days was demonstrated by H&E staining. Scale bar: 100 *μ*m. (b) The numbers of the hierarchical follicles (with diameters over 12 mm) were compared in hens aged 150, 280, and 580 days (*n* = 10).

The H&E staining of ovarian tissues from D90, D150, D280, and D580 hens showed that the number of growing follicles at D580 was markedly lower than that in other three groups (Figure 1(a)). Ten healthy hens from each group of laying hens were selected randomly for evaluating laying performance. The results showed that there was a sharp increase in hierarchical follicle number from the D150 hens to the D280 hens. However, a marked decline appeared from the D280 hens to the D580 hens (Figure 1(b)).

### 3.2. Age-Related Changes in Ovarian Antioxidant Capacity

**Figure 2:**
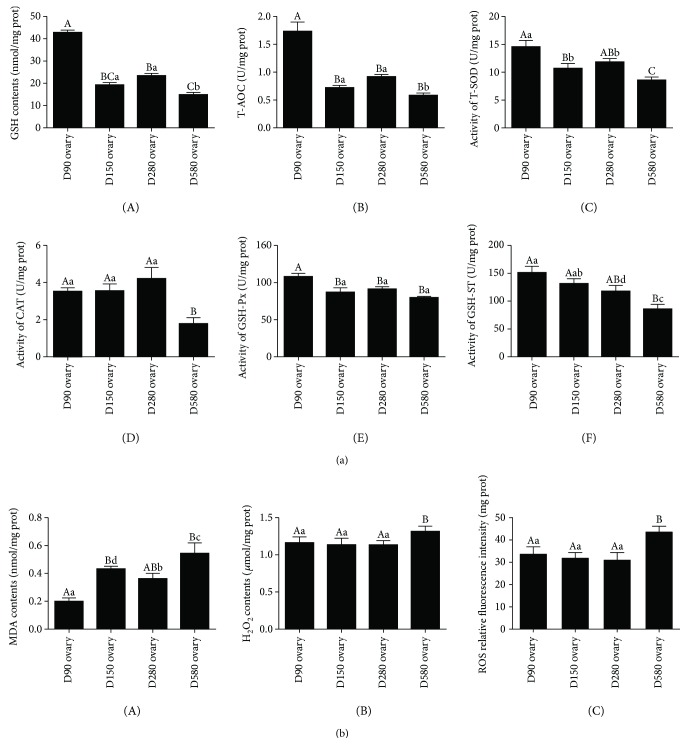
Ovarian antioxidant and oxidant levels in hens at different ages. (a) Ovarian antioxidant levels in hens at different ages. Levels of GSH A, T-AOC B, T-SOD C, CAT D, GSH-Px E, and GSH-ST F were determined in the ovary at the age of 90, 150, 280, and 580 days. (b) Ovarian antioxidant levels in hens at different ages. Levels of MDA A, H_2_O_2_ B, and ROS C were determined in the ovary at the age of 90, 150, 280, and 580 days. Data were expressed as the means ± s.e. (*n* = 10). Different capital and lowercase letters indicate a very significant difference (*P* < 0.01) and significant difference (*P* < 0.05), respectively.

Levels of GSH, T-AOC, and the activity of T-SOD in D580 ovary were significantly lower than those in other three stages, and a significant decrease was observed in ovaries from D90 pullets to laying hens. No marked difference was detected between D150 and D280 ovaries (Figure 2(a), A–C). There was no considerable difference in the activities of CAT among D90, D150, and D280 ovaries; however, there was a sharp decrease in D580 ovary compared with that in D280 ovary (Figure 2(a), D). The activity of GSH-Px in D90 ovary was significantly higher than that in other three stages, and no considerable difference was found among D150, D280, and D580 ovaries (Figure 2(a), E). The activity of GSH-ST in D580 ovary was markedly lower than that in other three stages, and D280 ovary was significantly lower than D90 ovary, but there was no significant difference between D90 and D150 ovaries and D150 and D280 ovaries (Figure 2(a), F). Meanwhile, the MDA contents in the ovaries increased significantly from D90 to D580, but no considerable difference was detected in MDA levels between D150 and D280 ovaries (Figure 2(b), A).The H_2_O_2_ contents (Figure 2(b), B) in the ovaries of D580 were markedly higher than those in other three stages. As illustrated in Figure 2, C, the ROS level in D580 ovarian tissues was significantly higher than that in other three stages. No significant difference was detected in ROS levels among D90, D150, and D280 ovaries.

**Figure 3:**
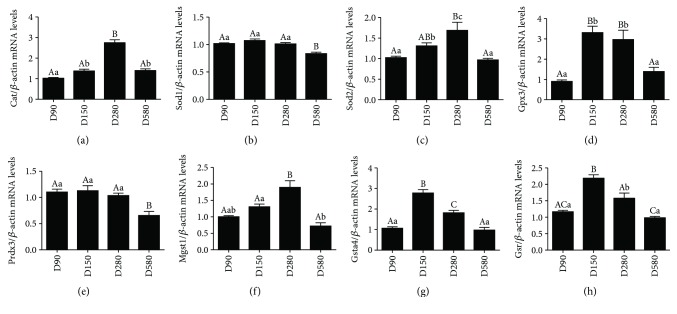
Transcription levels of genes related to ovary antioxidant capacity. Levels of *Cat* (a), *Sod1* (b), *Sod2* (c), *Gpx3* (d), *Prdx3* (e), *Mgst1* (f), *Gsta4* (g), and *Gsr* (h) mRNAs in ovaries of hens aged 90, 150, 280, and 580 days. Data were expressed as the means ± s.e. The relative abundance of each transcript was normalized to a *β*-actin and expressed as fold changes over D90 pullets. Different capital and lowercase letters indicate a very significant difference (*P* < 0.01) and significant difference (*P* < 0.05), respectively.

The results of age-related changes in the expression of ovarian antioxidant genes are shown in Figure 3. The mRNA abundance of *Cat* in hen ovaries increased significantly from D90 to D150 and reached the highest level at D280 while decreased sharply at D580 (Figure 3(a)). The mRNA levels of *Sod1* and *Prdx3* in D580 ovary were significantly lower than those in other three stages, and no considerable difference was detected among D90, D150, and D580 ovaries (Figures 3(b) and 3(e)). The mRNA levels of *Sod2*, *Gpx3*, *Mgst1*, *Gsta4*, and *Gsr* in the ovaries first increased from D90 to D150, then decreased from D280 to D580 (Figures 3(c), 3(d), and 3(f)–3(h)). Interestingly, the expression of *Gpx3*, *Gsta4*, and *Gsr* mRNAs in the ovary reached at a maximum at D150 while *Sod2* and *Mgst1* did at D280. The above results indicated that decreased ovarian antioxidant capacity and increased oxidative stress appeared during the aging process. Therefore, the screening for putative oxidative substances is particularly urgent for attenuating the oxidative stress in the aging process for poultry production.

### 3.3. Effects of GSPE on the Morphological Changes of the D-gal-Induced Aging Ovarian Tissues

**Figure 4:**
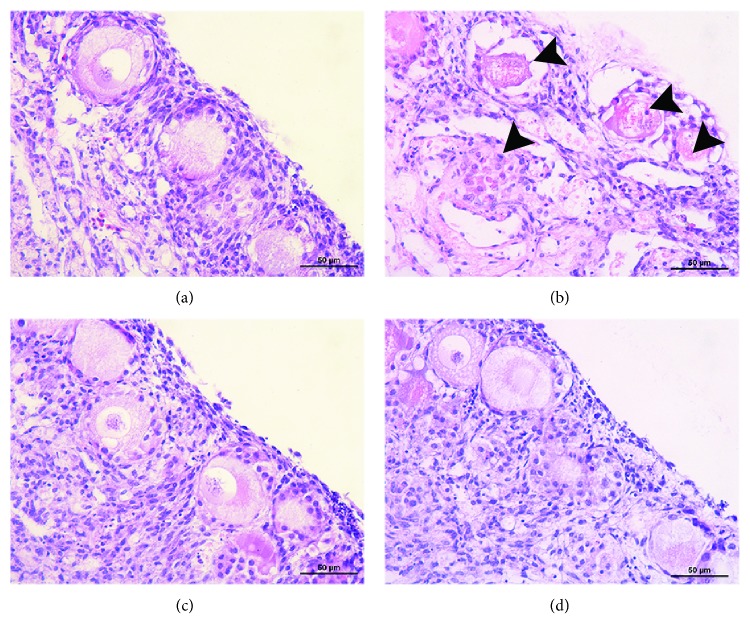
Effects of GSPE on D-gal-induced morphological changes of ovarian tissues. Representative morphology of ovarian tissues after 72 h of culture in the control group (a), D-gal group (b), GSPE group (c), and D-gal + GSPE group (d) by H&E staining. Scale bar: 50 *μ*m. Black arrow heads: apoptotic cells of the growing follicles.

Treatment of ovarian tissues with 2.5 mg/mL D-gal for 72 h markedly induced the damage of the granulosa cells, and the shape of growing follicles was also changed as the granulosa cells were arranged loosely and irregularly (Figures 4(a) and 4(b)). These adverse changes of the growing follicles and granulosa cells were alleviated by the combined treatment of GSPE while GSPE itself showed no obvious effect on growing follicles and granulosa cells (Figures 4(c) and 4(d)). These data indicated that D-gal-induced impair of the growing follicles structure can be reversed by GSPE.

### 3.4. Effects of GSPE on the Ultrastructural Changes of the Granulosa Cells in the D-gal-Induced Aging Ovarian Tissues

**Figure 5:**
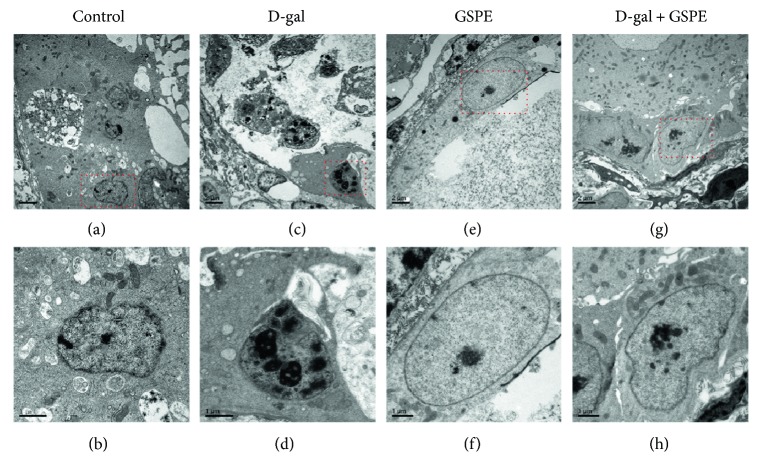
Effects of GSPE on D-gal-induced ultrastructural changes of granulosa cells. The ultramicrostructure of granulosa cells in ovarian tissues cultured for 72 h in the control group (a, b), D-gal group (c, d), GSPE group (e, f), and D-gal + GSPE group from TEM. (b, d, f, and h) are higher magnifications of the red square from (a, c, e, and g), respectively. Scale bar: 2 *μ*m (a, c, e, and g); 1 *μ*m (b, d, f, and h).

Through TEM observation, no significant difference was found in the morphology of the mitochondria, endoplasmic reticulum, and Golgi complex among four groups. However, in the D-gal group, the nucleus chromatin was condensed into clumps of different sizes while they were distributed normally in the control and GSPE groups. Meanwhile, after treatment of the ovarian tissues with GSPE and D-gal simultaneously for 72 h, the phenomenon of nucleus chromatin condensation was alleviated (Figure 5). These results indicated that GSPE could partially reverse the D-gal-induced nucleus chromatin condensation in the granulosa cells.

### 3.5. Effects of GSPE on ROS Levels in the D-gal-Induced Aging Ovarian Tissues

**Figure 6:**
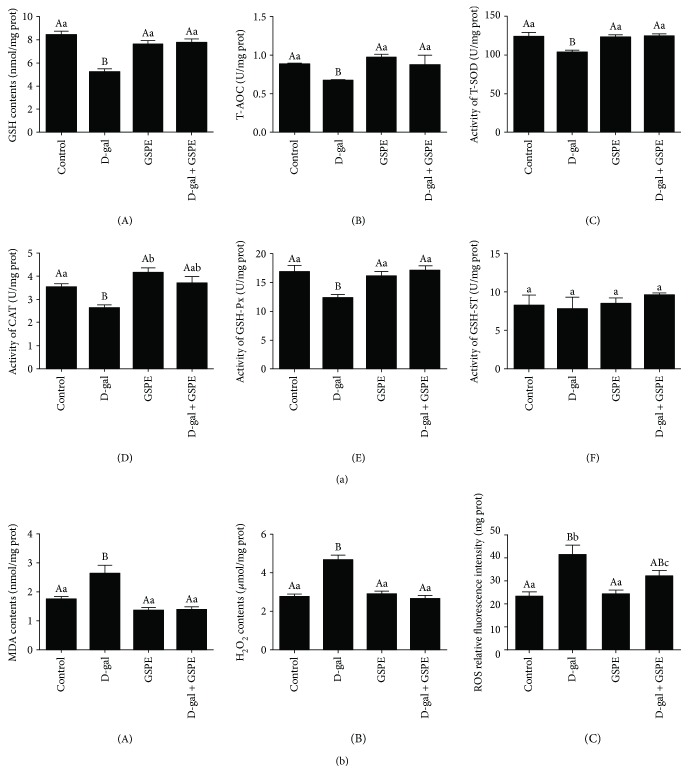
Effects of GSPE on decreased antioxidant status in D-gal-induced aging ovarian tissues. Antioxidase levels ((a) A: GSH, B:T-AOC, C: T-SOD, D: CAT, E: GSH-Px, and F: GSH-ST), oxidative products ((b) A: MDA and B: H_2_O_2_), and ROS levels (c) in the ovarian tissues after 72 h of GSPE treatment. Data were expressed as the means ± s.e. Different capital and lowercase letters indicate a very significant difference (*P* < 0.01) and significant difference (*P* < 0.05), respectively.

To confirm the effects of D-gal alone or combined with GSPE on oxidative stress in ovarian tissues, the levels of ROS in ovarian tissues from four treatment groups were determined. After treatment with D-gal alone for 72 h, the ROS level was significantly elevated compared with the control group. When GSPE was supplemented concurrently, ROS level dropped markedly but was not normalized to the level of the control group. No significant difference was detected in ROS levels between the control group and GSPE group (Figure 6(b), C). These results suggested that D-gal significantly increased the generation of ROS in ovarian tissues, and this increase could be attenuated by concurrent GSPE supplement.

### 3.6. Effects of GSPE on the Antioxidant Capacity Decline in the D-gal-Induced Aging Ovarian Tissues

In order to clarify the effects of D-gal alone or combined with GSPE on the antioxidant capacity of ovarian tissues, the activities of antioxidant enzymes as well as the contents of MDA and H_2_O_2_ in ovarian tissues from four groups were measured. The results showed that the GSH contents and the T-AOC were significantly decreased in the ovarian tissues after treatment with D-gal compared with that in the control group (Figures 6(a), A and B). Meanwhile, the activities of T-SOD, CAT, and GSH-Px were significantly decreased after D-gal treatment for 72 h. These descending trends were prevented by simultaneous administration of GSPE. There was no significant difference in GSH-ST activity among four groups. The activities of the T-SOD and GSH-Px, the levels of GSH, and the T-AOC were not changed after treatment with GSPE alone (Figure 6(a), C–E). Interestingly, the activities of CAT in the GSPE group and D-gal + GSPE group were higher than those in the control group. The MDA and H_2_O_2_ contents in the D-gal group were both markedly higher than those in other three groups. No significant difference was found in the contents of MDA and H_2_O_2_ among the control, GSPE, and D-gal + GSPE groups (Figure 6(b), A and B). These results indicated that the antioxidant capacity of ovarian tissues decreased after D-gal treatment, and GSPE could restore this decrease.

### 3.7. Effects of GSPE on the Changes of Antioxidant Gene Expression in the D-gal-Induced Aging Ovarian Tissues

**Figure 7:**
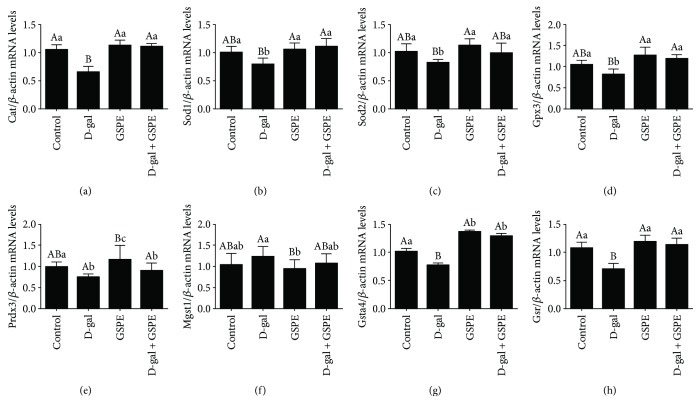
Effects of GSPE on the downregulation of the antioxidant gene expression in D-gal-induced aging ovarian tissues. Transcription levels of *Cat* (a), *Sod1* (b), *Sod2* (c), *Gpx3* (d), *Prdx3* (e), *Mgst1* (f), *Gsta4* (g), and *Gsr* (h) in ovarian tissues from the control group, D-gal group, GSPE group, and D-gal + GSPE group were determined after 72 h treatment. Data were expressed as the means ± s.e. Different capital and lowercase letters indicate a very significant difference (*P* < 0.01) and significant difference (*P* < 0.05), respectively.

The results from qRT-PCR showed that D-gal significantly decreased the expression of *Cat*, *Sod1*, *Sod2*, *Gsta4*, and *Gsr* mRNAs (Figures 7(a)–7(c), 3(g), and 3(h)), and these changes were normalized by combined GSPE administration. However, D-gal decreased the expression of *Prdx3* mRNA, but this decrease was not normalized by GSPE treatment (Figure 7(d)). Interestingly, the mRNA level of *Mgst1* was increased after D-gal treatment but decreased in the GSPE group (Figure 7(f)). These results suggested that GSPE could partially rescue the changes of antioxidant gene expression in D-gal-induced aging ovarian tissues.

### 3.8. Effects of GSPE on the D-gal-Induced Decline of Cell Proliferation

**Figure 8:**
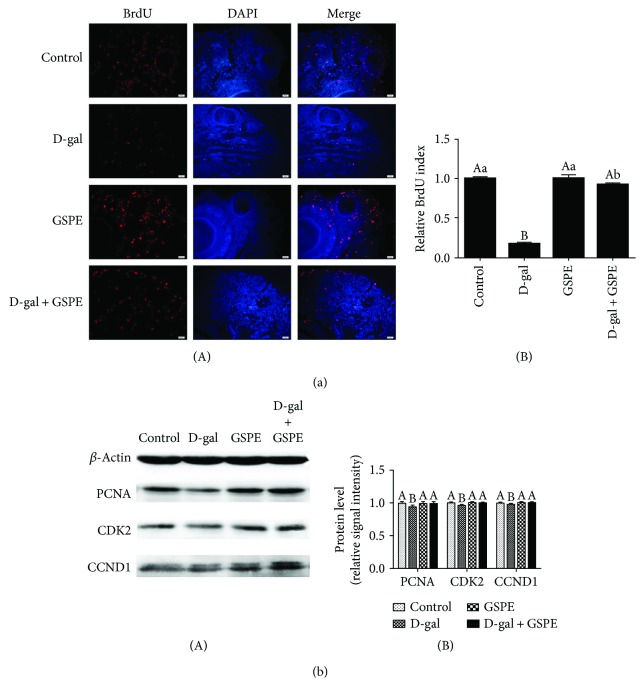
Effects of GSPE on D-gal-induced decline of cell proliferation. (a) After the incorporation with BrdU for 24 h, the BrdU-positive cells were compared in the control, D-gal, GSPE, and D-gal + GSPE groups. Scale bar: 20 *μ*m. (b) Relative expression of proteins related to cell proliferation. Data were expressed as the means ± s.e. Different capital and lowercase letters indicate a very significant difference (*P* < 0.01) and significant difference (*P* < 0.05), respectively.

Treatment of ovarian tissues with D-gal for 72 h manifested a dose-dependent inhibition of ovarian somatic cell proliferation (Supplementary Figures available here). D-gal treatment decreased the BrdU labeling rates in the ovarian somatic cells. GSPE treatment alone did not change the BrdU labeling rates. Consistent with the expectation, the decline of BrdU labeling rate that was induced by D-gal was inhibited by GSPE (Figure 8(a)). Moreover, D-gal significantly downregulated the expression of PCNA, CDK2, and CCND1, but these changes were normalized by GSPE supplement (Figure 8(b)). These data suggested that GSPE may inhibit the D-gal-induced decline of ovarian somatic cell proliferation.

### 3.9. Effects of GSPE on the D-gal-Induced Ovarian Cell Apoptosis

**Figure 9:**
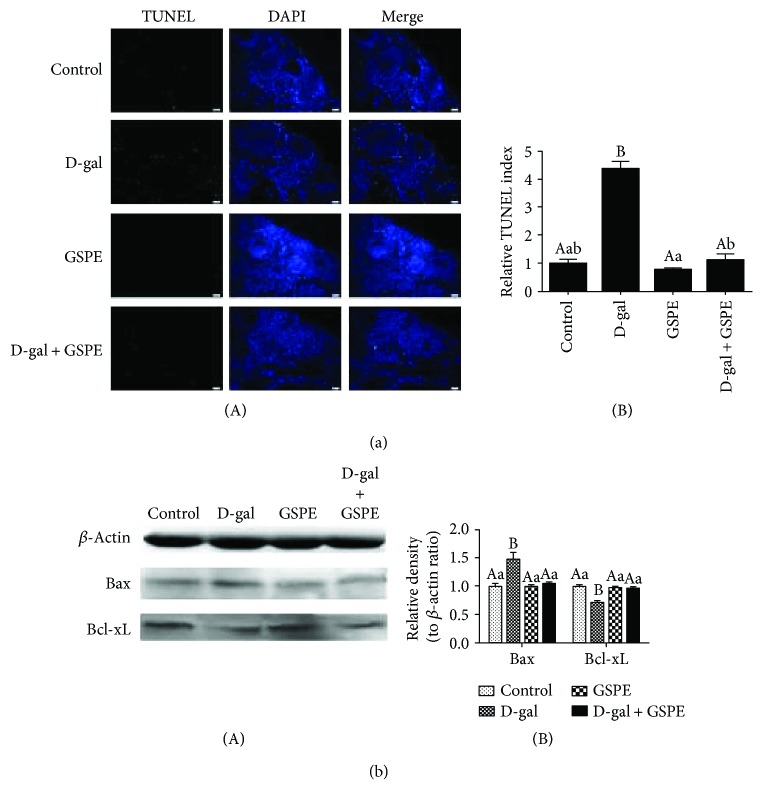
Effects of GSPE on D-gal-induced increase of cell apoptosis. (a) Ovarian cell apoptosis was determined by TUNEL assay. Scale bar: 20 *μ*m. (b) Relative expression of proteins related to proapoptosis (Bax) and antiapoptosis (Bcl-xL). Data were expressed as the means ± s.e. Different capital and lowercase letters indicate a very significant difference (*P* < 0.01) and significant difference (*P* < 0.05), respectively.

The results from TUNEL assay showed that the percentage of TUNEL-positive cells in the D-gal group was higher than that in the control group. Compared with the D-gal-treated group, the percentage of TUNEL-positive cells was significantly reduced in the D-gal + GSPE group (Figure 9(a)). Western blot analysis of the apoptosis-related proteins showed that the expression of Bax was significantly increased while the expression level of Bcl-xL was decreased in ovarian tissues from the D-gal group compared with the control group. However, compared with the D-gal-treated group, the expression of Bax was decreased and the expression of Bcl-xL was increased in the D-gal + GSPE group (Figure 9(b)).

### 3.10. GSPE Protected Ovaries from Oxidative Stress In Vitro during Natural Aging Process

**Figure 10:**
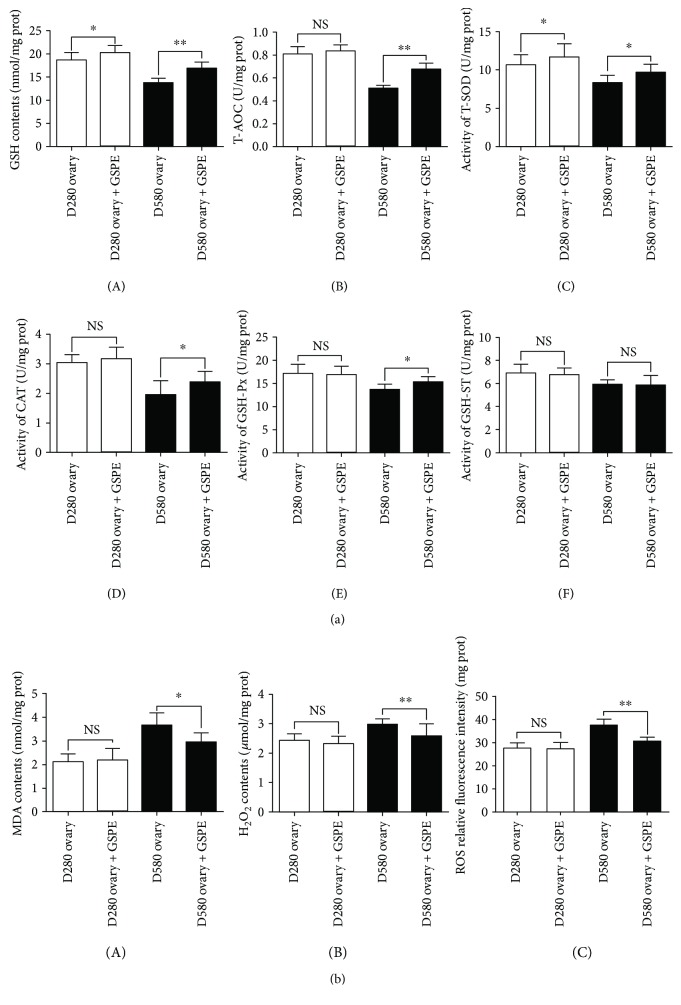
Effects of GSPE on antioxidant capacity in ovarian tissues of D280 and D580 *in vitro*. Antioxidase (a) (A) GSH, (B) T-AOC, (C) T-SOD, (D) CAT, (E) GSH-Px, and (F) GSH-ST and oxidative levels (b) produce (A) MDA, (B) H_2_O_2_, and (C) ROS levels in ovary after 72 h of GSPE treatment. Data were expressed as the means ± s.e. ^∗^ *P* < 0.05 and ^∗∗^ *P* < 0.01.

**Figure 11:**
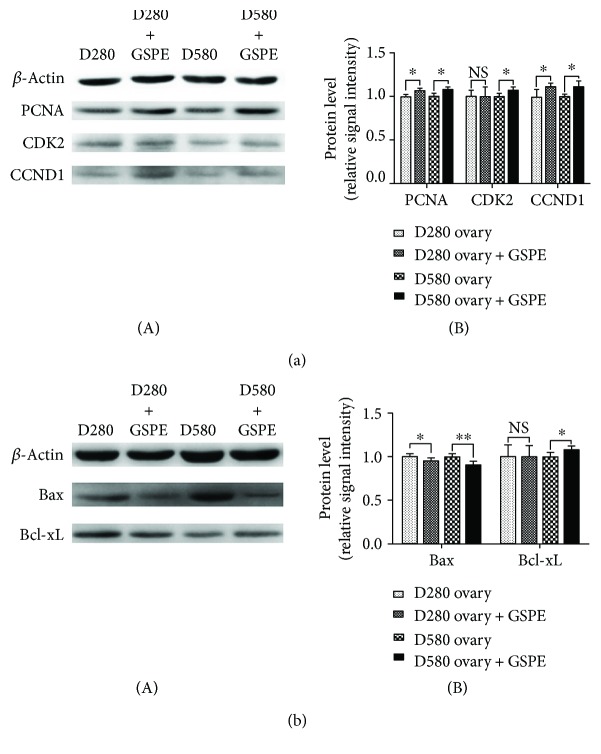
Effects of GSPE on cell proliferation and apoptosis in the ovarian tissues of D280 and D580 *in vitro*. (a) Relative expression of proteins related to cell proliferation. (b) Relative expression of proapoptotic protein (Bax) and antiapoptotic protein (Bcl-xL). Data were expressed as the means ± s.e. ^∗^ *P* < 0.05 and ^∗∗^ *P* < 0.01.

To evaluate whether treatment with GSPE was capable of protecting natural aging ovarian tissues from oxidative stress, D280 and D580 ovarian tissues were treated with/without GSPE for 72 h in culture. Then the antioxidant capacity, cell proliferation, and apoptosis of ovarian tissues in each group were determined (Figures 10 and 11).

After treatment with GSPE for 72 h, the GSH contents and the activity of T-SOD in D280 and D580 ovarian tissues were both significantly increased (Figure 10(a), A and C). Treatment with GSPE could increase the T-AOC and activate CAT and GSH-Px in ovarian tissues at D580 but not at D280 (Figure 10(a), B, D, and E). However, the activity of GSH-ST in D280 and D580 ovarian tissues was not changed significantly after 72 h of GSPE treatment (Figure 10(a), F). Treatment with GSPE could decrease MDA contents, H_2_O_2_ contents, and ROS levels in the cultured ovarian tissues at D580 but not at D280 (Figure 10(b)).

The results by Western blot analysis showed that GSPE treatment increased the expression of PCNA and CCND1 in ovarian tissues of D280 and D580 and the expression of CDK2 (Figure 11(a)) and Bcl-xL (Figure 11(b)) in ovarian tissues of D580. Furthermore, the expressions of Bax in the ovaries of D280 and D580 were both significantly decreased (Figure 11(b)) after 72 h treatment of GSPE.

## 4. Discussion

Poultry meat and eggs are rich sources of animal protein in human daily life. In order to satisfy the demands for meat and eggs, more and more highly productive hens have been bred. However, at the end of the first year laying cycle in most highly productive laying hens, a sharp decrease in egg production appears and inevitably reduces the commercial value of the laying hens. Therefore, it is crucial to elucidate the mechanisms underlying reproductive aging in the laying hens. It has been demonstrated that the ovarian lifespan is the main determinant of female reproductive function [31]. Female reproductive aging includes the reduction in ovarian follicle reserve and the decline in oocyte quality [4, 5]. One of the major factors that caused ovarian aging is the oxidative stress that is elicited by the gradual accumulation of ROS and the decline of antioxidants in the ovary during the aging process [15].

Growing evidences demonstrated that the supplementation of antioxidants is an efficient measure to reduce the oxidative stress in the ovary [32, 33]. Among the dairy plant antioxidants, GSPE is an extract from red grape seeds and skin. It has been shown to be a potent antioxidant and effective scavenger of free radicals [34]. Recent studies have shown that GSPE could alleviate arsenic and cisplatin-induced oxidative stress in the testes of rodents [35, 36]. Our previous study demonstrated that GSPE alleviated Cd-induced meiotic abnormalities of the ovarian tissues in chicken embryos [37]. In the present study, we investigated the effects of aging on ovary antioxidant status via comparing ovarian histology, antioxidants, and oxidants in the ovary and the expression levels of genes related to antioxidant capacity in ovaries of hens in different laying stages. Then, we investigated the potential of GSPE to alleviate the oxidative stress in the D-gal-induced aging ovaries. Furthermore, we verified the protective effects of GSPE against oxidative stress on natural aging ovarian tissues. Our results validated that the antioxidant status in the ovary of laying hens increases from pullets to laying hens and then decreases sharply with aging. Meanwhile, our results showed that GSPE supplementation effectively alleviated the oxidative stress in aging ovaries by restoring the homeostasis between apoptosis and proliferation.

The reproductive aging in hens is associated with an increase in atresia of the slow-growing follicles, plus a decreased rate of follicle selection into the preovulatory hierarchy [38]. In this study, there was a sharp increase in hierarchical follicle numbers from early laying hens (D150) to peak laying hens (D280); however, a marked decline appeared from D280 to later laying hens (D580). These data are in accordance with a previous study that there is a moderately rapid decline in fertility in chickens during aging process [39].

Ovulation occurs by a mechanism similar to inflammatory reaction, which is accompanied by oxidative stress [40]. Increases of oxidative damage and decreases of antioxidant gene expression may play a role in the decline of ovarian function in the aging mouse [41]. In the highly productive hens, near-daily incidence of ovulation accelerates the accumulation of ROS in ovaries. Decreased expression of *Sod1* and *Gsr* was detected in the germinal vesicle-intact or ovulated oocytes from the old mice compared with the younger mice [42]. In the hexavalent chromium- (CrVI-) induced oxidative stress model, expression levels of *Gsta1*, *Gsta2*, and *Gsta4* in the rat ovary, kidney, and liver were all decreased, compared with the normal condition [32, 43]. Our results showed that ovarian levels of GSH, T-AOC, T-SOD, CAT, and GSH-ST decreased significantly while MDA, H_2_O_2_, and ROS levels increased during aging process. In addition, the mRNA levels of most antioxidant genes first increased from pullets to sexually mature hens and then decreased sharply with approaching aging. Similar findings were noticed by Lim and Luderer [41] who found that the expression of the antioxidant genes in mouse ovary was decreased with aging. After treatment with D-gal for 72 h, the contents of GSH, the T-AOC, and the activities of T-SOD, CAT, and GSH-Px decreased significantly accompanied with the increased ROS, MDA, and H_2_O_2_ levels in the ovarian tissues. We observed that GSPE significantly reduced the levels of ROS, MDA, and H_2_O_2_ in D-gal-induced aging ovaries and natural aging ovaries *in vitro*. Meanwhile, the decline in the activities of the antioxidant enzymes, the levels of oxidants, and the expression levels of antioxidant genes in D-gal-induced aging ovarian tissues and natural aging ovarian tissues were inhibited after the supplementation of GSPE. Unexpectedly, D-gal or GSPE treatment did not influence the morphology of mitochondrial, endoplasmic reticulum and Golgi complex in the living granulosa cells. We assumed that the time of treatment may not be long enough for D-gal to elicit damage of the organelles after only 72 h in this study.

Apoptosis is a critical process in the growth, development, and homeostasis of diverse organs [44]. However, excessive apoptosis rates may cause organ dysfunction [45]. Previous studies demonstrated that the apoptotic pathways could be activated by elevated intracellular ROS production [46, 47]. Bcl-xL and Bax are two important proteins of the Bcl-2 family. The Bcl-xL is antiapoptotic while the Bax is proapoptotic [48], and Bcl-xL is a potent inhibitor of Bax [49]. The decline in the Bax/Bcl-xL ratio in circulating mononuclear cells led to decreased cell apoptosis [50]. Our results indicated that the D-gal treatment increased the expression level of Bax and decreased the expression level of Bcl-xL. Furthermore, a significant increase in the TUNEL-positive cells was observed in the D-gal treated ovaries. However, GSPE supplement partly reversed these changes via increasing the expression of Bcl-xL and decreasing the expression of Bax. Meanwhile, compared with those in the D-gal-treated group, the TUNEL-positive cells in the D-gal + GSPE group was significantly decreased. BrdU incorporation and Western blot analysis demonstrated that D-gal treatment significantly inhibited the proliferation of ovarian somatic cells. However, these changes were all attenuated by GSPE supplement.

The results of verification experiments showed that *in vitro*, GSPE could improve the antioxidant capacity in the aging ovarian tissues and maintain the homeostasis between cell proliferation and apoptosis, finally retarding the ovarian aging.

## 5. Conclusion

This study demonstrated that the ovarian antioxidant status decreased in the natural aging process as a result of decreased activities of the antioxidase and transcription levels of antioxidant genes as well as increased oxidants levels in the hens. In the D-gal-induced ovarian aging model, the antioxidant status of ovarian tissues and ovarian somatic cell proliferation were decreased while the cell apoptosis rate was increased. Meanwhile, D-gal treatment impaired the morphology of the growing follicles and induced nucleus chromatin condensation in the granulosa cells. Simultaneous GSPE treatment decreased the oxidative stress, alleviated the inhibition of aging on ovarian somatic cell proliferation, and decreased cell apoptosis in the D-gal-induced as well as natural aging ovarian tissues *in vitro*. Overall, this study provided first-hand evidence of potential utilization of GSPE in the protection of oxidative stress in the aging ovaries of the laying hens, and the D-gal-induced ovarian aging model may represent a prospective method for antiaging pharmacological screening.

## Figures and Tables

**Table 1:** Primers for PCR analysis.

Gene name	Accession number	Primer sequence (5′-3′)	Product size (bp)
*Cat*	NM_001031215.2	TCAGGAGATGTGCAGCGTTT	109
		TCTTACACAGCCTTTGGCGT	
*Sod1*	NM_205064.1	GGCAATGTGACTGCAAAGGG	133
		CCCCTCTACCCAGGTCATCA	
*Sod2*	NM_204211.1	TCCTGACCTGCCTTACGACT	139
		TGCCAGCGCCTCTTTGTATT	
*Gpx3*	NM_001163232.2	AGGAGTACATCCCCTTCCGA	124
		TAGGGCCCCAGCTCATTTTG	
*Prdx3*	NM_426543.5	TGGATAAATACCCCGCGCAA	126
		TCTCAGTGCAATGCCAGGTC	
*Mgst1*	NM_001135550.1	GGCATTTGCCAACCCAGAAG	116
		CAAGGTCATTCAGGTGGCCT	
*Gsta4*	NM_204818.2	GCAGAGCCATCCTCAGCTAC	150
		CCTTTGCCTCAGGTGGAGAG	
*Gsr*	XM_015276627.1	TCCTGACTACGGCTTCGAGA	150
		AACTTGCCGTAACCACGGAT	
*β*-Actin	NM_205518	ACACCCACACCCCTGTGATGAA	136
		TGCTGCTGACACCTTCACCATTC	

## Supplementary Materials

Supplementary Materials Figure 1: Effects of D-gal at different doses on ovarian tissue morphology. Scale bar: 50 *μ*m. Representative morphology of ovarian tissues after 72 h of culture in the control, 1.25 mg/mL D-gal, 2.5 mg/mL D-gal, and 5 mg/mL D-gal groups which were performed by H&E staining. Scale bar: 50 *μ*m. Figure 2: Effects of D-gal at different doses on ovarian somatic cell proliferation. After the incorporation with BrdU for 24 h, the BrdU-positive cells were compared in the control, 1.25 mg/mL D-gal, 2.5 mg/mL D-gal, and 5 mg/mL D-gal groups. Scale bar: 20 *μ*m. DAPI staining was performed to stain the nucleus. Figure 3: Effects of D-gal at different doses on ovarian somatic cell proliferation. After the incorporation with BrdU for 24 h, the BrdU-positive cells were compared in the control, 1.25 mg/mL D-gal, 2.5 mg/mL D-gal, and 5 mg/mL D-gal groups. Scale bar: 20 *μ*m. DAPI staining was performed to stain the nucleus. Figure 4: Protective effects of lycopene at different doses on D-gal-induced ovarian morphology change were determined by H&E staining. Scale bar: 50 *μ*m. Figure 5: Protective effects of GSPE at different doses on D-gal-induced ovarian cell proliferation decline which were determined by BrdU staining. Scale bar: 20 *μ*m. DAPI staining was performed to stain the nucleus. Figure 6: Protective effects of GSPE at different doses on D-gal-induced ovarian cell proliferation decline which were determined by BrdU staining. Scale bar: 20 *μ*m. DAPI staining was performed to stain the nucleus.
